# Effect of prey size and structural complexity on the functional response in a nematode- nematode system

**DOI:** 10.1038/s41598-019-42213-x

**Published:** 2019-04-05

**Authors:** Bianca Kreuzinger-Janik, Henrike Brüchner- Hüttemann, Walter Traunspurger

**Affiliations:** Bielefeld university, Animal Ecology, Konsequenz 45, 33615 Bielefeld, Germany

## Abstract

The functional response of a predatory nematode and the influence of different prey sizes and habitat structure on the concerning parameters were analyzed. We hypothesized that the handling of small prey would be less time-consuming, whereas feeding on larger prey would be more efficient. Therefore, type II functional response curves were expected for large prey and a trend towards type III curves for small prey. We expected the introduction of prey refuges to shift the functional response curves from hyperbolic to sigmoidal and that the effect would be even more pronounced with smaller prey. *P. muscorum* consumed large amounts of small and large *C. elegans*, with daily *per capita* ingestion of prey reaching a maximum of 19.8 µg fresh weight, which corresponds to 4.8 times the predator’s biomass. Regardless of prey size and habitat structure, *P. muscorum* exhibit a type III functional response. Overall, the allometric effect of prey size had a greater effect on the predator’s functional response than did the addition of substrate, presumably due to the similar body shape and mobility of the two nematode species. Our results demonstrate that individual factors such as feeding behavior are important determinants of functional responses and therefore of ecosystem stability.

## Introduction

The quantitative characteristics of consumer-resource interactions determine our understanding of the stability of population dynamics, complex food webs and ecosystem function. These interactions are based on mechanisms, which can be experimentally investigated in functional-response experiments by analyzing the rate of ingestion of a consumer as a function of food density^1^. Three different types of functional responses were categorized by Holling^2^, depending on the curve progression. A type I functional response describes a linear relation between the predation rate and prey density up to a threshold and is typical of filter feeders (reviewed by^3^). A type II functional response is characterized by a hyperbolic curve and describes an increase in the amount of prey ingested with increasing prey density, until saturation of prey ingestion occurs. If the increase in the number of prey consumed decelerates when only few prey organisms are available, the shape of the curve becomes sigmoidal, indicative of a type III functional response and a switching or learning behavior of the predator^4–6^. A type II functional response was long expected to be the most common^7–9^, but more recent studies have clearly demonstrated the type III functional responses of many species^10–16^. Moreover, type III responses are not necessarily related to multi-prey environments, in contrast to previous reports^5,17^. In addition, there is strong evidence that functional response parameters (e.g. handling time, search coefficient) depend on the body masses of predator and prey^13,14,18,19^, the environmental temperature^20,21^, and habitat complexity [e.g.^22,23^], in addition to factors such as prey defenses, prey type and prey morphology^24,25^.

Nematodes are the most abundant metazoan group within the meiofauna, reaching maximum densities >10^6^ individuals per m² in the sediment and periphyton of stony hard-substrates of freshwater habitats^26,27^. They colonize all types of habitats and climatic zones and establish a permanent standing stock in the course of the year, comprising many different species^28–31^. Nematodes are exploited as prey, with a top-down influence on species composition and population dynamics exerted by their predators, including vertebrates such as juvenile fish^32,33^, and both macro- and meiofaunal invertebrates, such as copepods, tardigrades, turbellarians and chironomid larvae^16,22,34,35^. However, nematodes represent an important link between the microbenthos and macrobenthos of freshwater ecosystems^27,36,37^. With their diverse range of feeding types, they are able to parasite plants and animals, feed as free-living species on micro-algae, fungi, and bacteria and prey on other invertebrates, including other nematode species, as well as protozoans (reviewed by^38^). Yet, the potential impact of predatory nematodes on meiofauna and especially on the nematode community has been poorly documented (but see^39,40^).

Most studies of predatory nematodes have focused on agricultural aspects and the potential of the respective species as biocontrol agents (reviewed by^41^) whereas few studies have examined the importance of nematode predators as regulatory forces in food webs. To our knowledge, only one study quantitatively assessed the functional response of predatory nematodes, using two estuarine nematode species as predators^42^).

Habitat complexity may be a crucial factor in the feeding success of predatory nematodes living in the interstices of sediments or plant material (e.g. moss, macrophytes). A more structurally complex habitat may be advantageous for potential prey, by offering hiding places and thereby increasing their survival probability^15,43,44^. Conversely, habitat complexity may promote prey mortality due to higher foraging efficiency of the predator^45^. A recent study^46^ demonstrated that an increase in the amount of predator-free space causes a shift from a type II to a type III functional response, with effects on the stability of predator- prey dynamics based on an accelerated predation risk at low resource densities.

In the present study, we conducted for the first time a classic functional-response experiment with the common mononchid nematode *Prionchulus muscorum* as predator and two different size classes of *Caenorhabditis elegans* as prey to measure the predator feeding rate in relation to prey density. We hypothesized that the handling of small prey would be easier and less time-consuming for the predator, whereas feeding on larger prey would be more efficient, especially at low prey densities and higher search coefficients for prey of higher body mass. Therefore, a hyperbolically shaped functional response curve (type II) was expected for large prey and a trend towards a sigmoidal functional response curve (type III) for small prey.

In a second functional-response experiment, the conditions were the same as in the first but the substrate was altered to simulate habitat complexity. It was anticipated that the introduction of prey refuges would shift the shape of the functional response curves from hyperbolic (type II) to sigmoidal (type III) and that the effect would be even more pronounced when the predator was confronted with smaller prey. Consequently, capture rates would be negatively affected by habitat complexity, whereas handling times were expected to remain constant over all experimental trials using prey of the same size.

## Material and Methods

### Organisms

Individuals of the typical moss inhabiting large semiaquatic nematode species *Prionchulus muscorum* were extracted from terrestrial moss samples collected near Bielefeld University, Germany (52°02′16.1″N 8°29′26.5″E) using a modified Baermann funnel and used as predator. A sieve plate (594 cm², hole width 500 µm) was covered completely with moss and then placed on a funnel filled with filtered (10 µm) rainwater. After 24 h, the water in the funnel was filtered (10 µm) to obtain the organisms. Individuals of *P. muscorum* were selected individually using a binocular (Olympus SZ40, 40–100x magnification) and placed in a Petri dish filled with 2% agar covered with a thin layer of Volvic water. The body length and dry biomass of *P. muscorum* were 1623.97 ± 269.8 µm (mean ± SD, n = 264) and 1.149 ± 0.606 µg (mean ± SD, n = 264), respectively. The predators were starved for 3 days prior to the experiments.

Two size classes of the nematode species *Caenorhabditis elegans* were used as prey. *C. elegans* was chosen because this species represents in length and body shape a typical (semi-) aquatic nematode community and can be obtained in very high numbers from laboratory cultures which was a crucial factor to perform the functional response experiments simultaneously. The nematodes were cultivated by transferring pieces of nematode-containing agar onto fresh agar plates (0.85% agar, 0.125% peptone, 0.15% NaCl, 500 µl CaCl_2_ l^−1^, 500 µl MgSO_4_ l^−1^, 12.5 ml KH_2_PO_4_ l^−1^, and 500 µl cholesterol l^−1^) with a fresh lawn of *Escherichia coli* OP50^47^. After 7 days of incubation at 20 °C, the plates were rinsed with filtered (0.2 µm), autoclaved rainwater and the nematode suspension was then filtered sequentially through 20-µm, 10-µm and 5-µm sieves. *C. elegans* passing through the 20-µm sieve but retained on the 10-µm sieve had a mean body length of 383.9 ± 56.1 µm (mean ± SD, n = 50) and are hereafter referred to as “large prey”. *C. elegans* that passed through the 5-µm sieve (stage 1 juveniles^48^) had a mean body length of 308.9 ± 66.1 µm (mean ± SD, n = 50) and are hereafter referred to as “small prey”. The dry biomass of the large and the small prey was 0.017 ± 0.006 µg and 0.009 ± 0.004 µg (mean ± SD, n = 50), respectively.

### Measurements

The maximum body length and width of all tested individuals of *P. muscorum* and the lengths and widths of 50 small and large juvenile *C. elegans* were measured using a binocular (Olympus SZ40) and a stereomicroscope (Leica MZ 125) fitted with a camera (ProgRes C12plus). The images were processed using software ImageJ 1.48 (public domain).

The biomass was calculated according to Andrássy^49^ (Eq. 1):

1 $${{\rm{Biomass}}}_{(\mu g)}={{{\rm{width}}}_{(\mu m)}}^{2}\times {{\rm{length}}}_{(\mu m)}/1,\,600,\,000$$

assuming a nematode dry mass that was 25% of the fresh weight and a specific gravity of 1.13^50^.

### Functional-response experiments

The four sets of functional response experiments tested two size classes (small and large) of prey, with and without substrate, and were performed in open vials with a volume of 10 ml and a diameter of 20 mm (3.1 cm^2^ bottom area). All vials were filled with 1 ml of filtered (10 µm), autoclaved (121 °C, 20 min) rainwater and 1 ml of Volvic water. Half of the prepared vials were additionally filled with 0.04–0.05 g of dried, autoclaved moss (*Hypnum cupressiforme*) as substrate to add structural complexity and create a more natural 3D environment. The nematodes were added individually to the vials by mouth pipetting and allowed to acclimatize for 1 h before the start of the 4-h experiment, conducted at 20 °C (room temperature) and under low-light conditions. For each prey size class with and without substrate, 11 densities of *C. elegans* (5, 10, 15, 20, 30, 50, 75, 100, 150, 200, and 300) as prey and one specimen of *P. muscorum* as predator per vial were used. Additionally, a control treatment was conducted using prey at the same densities but without predator. Each prey density condition was replicated six times, both for the predator and the control (without predator), for the two size classes of *C. elegans* and with or without substrate, for a total of 528 samples (132 vials for each of the four treatments). All replicates of one treatment (predator and control) were placed randomly in a white box and tested at the same time. The experiment was started after carefully placing one predatory nematode in each vial. After 4 h, the nematodes were checked for viability. They were then heat-killed (1 h at 60 °C), stained with Rose Bengal and immediately counted at 45× magnification under a Leica S6E stereomicroscope.

The mean of the controls (n = 6) for each density was used to correct the initial density value. The difference between the corrected initial and counted final prey densities in the predator treatments was defined as the number of prey consumed by the predator. In some cases, this led to negative values, which were set to zero.

### Statistical analyses

Type II and type III functional responses can be distinguished based on the shape of the curves obtained by fitting polynomial logistic functions to proportional consumption data. A type II response is characterized by a negative first-order term (decreasing proportional consumption with increasing prey density), and a type III functional response by a positive first-order followed by a negative second-order term (increasing but then decreasing proportional consumption with increasing prey density)^51^. However, while this approach describes the general shape of the functional response, it is limited for particular mechanistic models. For this reason, we fitted flexible functional response models to allow a continuum of shapes, representing categorical type II to type III responses, and then compared the suitability of the models using the corrected Akaike information criterion (AICc), in which a lower AICc indicates a better fit.

A type II functional response of the predators was modeled using the appropriate equations, with prey depletion considered by integrating instantaneous consumption over time, and solving them using the ‘Lambert-W’ function^52^ (Eq. 2):

2 $${N}_{e}={N}_{0}[1-exp(a({N}_{e}h\,-\,T))]$$

Type III responses were modeled as described by Eq. 3^13,53^:

3 $${N}_{e}={N}_{0}[1\,-\,exp(b{{N}_{0}}^{q}({N}_{e}h\,-\,T))]$$

where *N*_*e*_ is the number of *C. elegans* consumed, *N*_0_ the number of prey presented, *a* the attack rate, *h* the processing time for a single prey item, *T* the total time available, *b* the capture coefficient and *q* the scaling exponent. When *q* = 0, the capture rates are constant with prey density, resulting in a decelerating hyperbolic type II functional response. For *q* > 0, the functional responses become increasingly sigmoidal (type III) and follow a power-law relationship with resource density. As in Pritchard *et al*.^54^, the data were nonparametrically bootstrapped (n = 2000) before a model using Eq. 2 or 3 was fitted. A type III functional response was assumed only when the scaling exponent *q* was significantly greater than zero. The resulting functional response curve was plotted together with the 95% confidence intervals. Estimates of the model parameters attack rate (*a*) and handling time (*h*) for type II functional responses, and the search coefficient (*b*), handling time (*h*), and scaling exponent (*q*) for flexible type III functional responses are shown.

All statistical analyses were carried out using R 3.4.3^55^. The results of the functional response experiments were analyzed using the R package *frair*^54,56^, which utilizes the maximum likelihood estimation within the *bbmle* package^57^.

## Results

**Figure 1:**
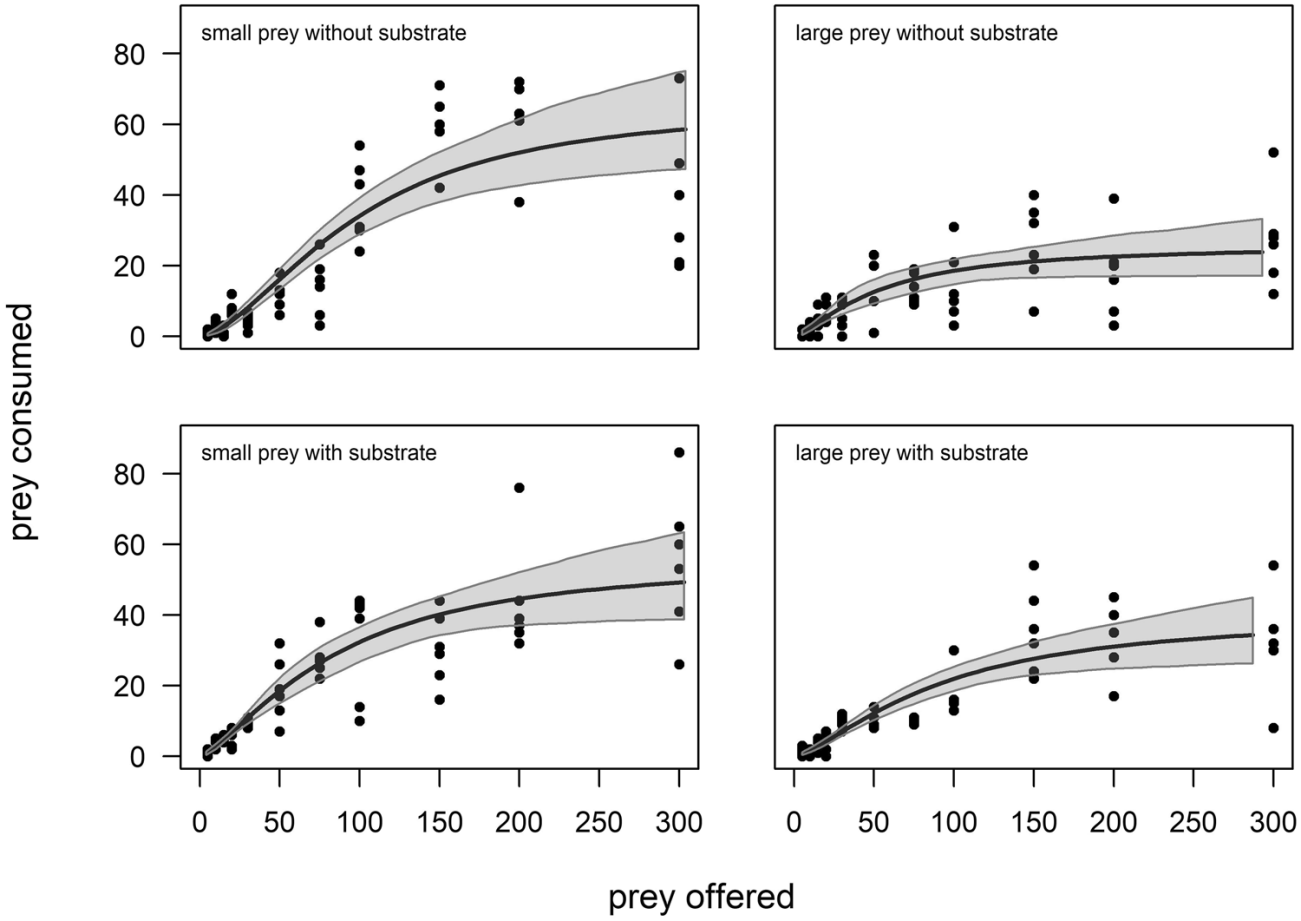
Number of small and large *Caenorhabditis elegans* consumed by *Prionchulus muscorum* within 4 h at offered nematode densities of 5–300 individuals, in the absence and presence of substrate. Data from all six replicates are shown. The curves represent the best-fitting functional response model (flexible type III functional response, see Eq. 3 in the text); 95% bootstrapped confidence intervals are shown as well. Parameter estimates are given in Table 2.

The maximum *per capita* ingestion of nematode prey per 4 h in the treatments without substrate occurred at a prey density of 300 nematodes, with 73 small and 52 large *C. elegans* individuals consumed in the respective treatments (Fig. 1). The maximum ingested biomass was therefore 2.409 µg (dry weight 0.681) and 3.172 µg (dry weight 0.896), respectively (accounting for up to 77% of the predators own biomass). Within the 4-h experiment, the number of consumed nematodes of either size class increased but only up to a density of 150 offered individuals. Thereafter, at higher prey densities, the mean intake remained constant for small and large prey: 54 ± 18 and 24 ± 13 ingested nematodes, respectively.

In the presence of the moss substratum, saturation occurred at a density of 200 offered prey individuals. Consumption reached a plateau at ~45 small and ~30 large nematodes (Fig. 1) and a maximum of 86 small and 54 large *C. elegans*, which occurred at a density of 300 offered prey items, were consumed. This corresponded to a maximum ingested biomass of 2.838 µg (dry weight 0.802) and 3.294 µg (dry weight 0.931), respectively, and up to 80% of the average weight estimated for *P. muscorum*.

Logistic regression analyses indicated that the functional response curves of *P. muscorum*, regardless of prey size and substrate, could be appropriately described by the categorical type II functional response model, since the first-order terms were all negative and significant (all p < 0.001, Table 1). Nevertheless, two- term logistic regressions also provided significantly positive first-order terms for small prey in the absence and large prey in the presence of substrate (both p < 0.01, Table 1), indicating a type III functional response in which the scaling exponent *q* is allowed to vary. A comparison of the two models using the AICc yielded consistently lower values for the flexible type III functional response model, even though it contained an extra parameter. Thus, type III was selected as the preferred model (Fig. 1).

**Table 1:** The results of logistic regressions for the selection of categorical type II or type III functional response models are shown, together with the corrected Akaike information criterion (AICc) values for fitted categorical type II (see Eq. 2 in the text) and flexible type III (Eq. 3) functional response models for treatments without and with substrate and using large vs. small *C. elegans* prey.

		*without substrate*		*with substrate*	
		small prey	large prey	small prey	large prey
logistic regression type II	1st term	−0.0032	−0.0049	−0.004	−0.004
	*P*	<0.001	<0.001	<0.001	<0.001
logistic regression type III	1st term	0.0153	−0.0095	−0.0053	0.0049
	*P*	<0.001	<0.001	<0.001	0.004
	2nd term	<−0.001	<−0.001	<−0.001	<−0.001
	*P*	<0.001	0.005	0.187	<0.001
AICc: type II model		773.957	584.44	552.957	484.796
AICc: flexible type III model		692.081	580.989	537.267	470.306

However, both models could be applied to each of the four experimental trials, yielding estimates for all parameters (Table 2). The attack rate was higher for small than for large prey, whereas the handling time increased with increasing prey size (Table 2, type II). In the absence of substrate, the search coefficient was lowest for small prey and highest for large prey but in the presence of substrate it reached a higher value for small than for large prey (Table 2, type III). The estimated handling time in the flexible type III functional response followed the same pattern as that in the categorical type II functional response, but the effect was less pronounced. For the experiment that included substrate, comparable scaling exponents were obtained for small and large *C. elegans*: 0.426 ± 0.11 and 0.438 ± 0.114, respectively. In the experiment without substrate, the scaling exponent was 0.739 ± 0.087 for small and 0.362 ± 0.166 for large prey (Table 2, type III). Table 3 shows the statistical output obtained in comparisons of the functional response parameters of the flexible model for the analysis of the influence of prey size [small vs. large and (small + substrate) vs. (large + substrate)] and habitat structure [small vs. (small + substrate) and large vs. (large + substrate)].

**Table 2:** Parameter estimates from categorical type II (see Eq. 2 in the text) and flexible (Eq. 3) functional responses for the treatments without and with substrate and large vs. small *C. elegans* prey. Data are the original maximum likelihood estimates ± SE and the p-values.

		*without substrate*		*with substrate*	
		small prey	large prey	small prey	large prey
type II (Eq. 2)	attack rate	2.686 ± 0.142 (p < 0.001)	2.343 ± 0.201 (p < 0.001)	3.104 ± 0.195 (p < 0.001)	1.893 ± 0.142 (p < 0.001)
	handling time	0.001 ±< 0.001 (p < 0.001)	0.005 ±< 0.001 (p < 0.001)	0.002 ±< 0.001 (p < 0.001)	0.003 ±< 0.001 (p < 0.001)
flexible (Eq. 3)	search coefficient	0.173 ± 0.059 (p = 0.003)	0.814 ± 0.402 (p = 0.043)	0.781 ± 0.285 (p = 0.006)	0.433 ± 0.172 (p = 0.012)
	handling time	0.003 ±< 0.001 (p < 0.001)	0.006 ± 0.001 (p < 0.001)	0.003 ±< 0.001 (p < 0.001)	0.004 ±< 0.001 (p < 0.001)
	scaling exponent	0.739 ± 0.087 (p < 0.001)	0.362 ± 0.166 (p = 0.029)	0.426 ± 0.11 (p < 0.001)	0.438 ± 0.114 (p < 0.001)

**Table 3:** Statistical output (p-values) obtained in comparisons of the search coefficients, handling times and scaling exponents resulting from a flexible (Eq. 3) functional response and depending on prey size and habitat structure.

small vs. large	search coefficient	handling time	scaling exponent
	p = 0.114	p < 0.001	p = 0.044
small + substrate vs. large + substrate	p = 0.297	p = 0.009	p = 0.938
small vs. small + substrate	p < 0.001	p = 0.189	p < 0.001
large vs. large + substrate	p = 0.43	p = 0.002	p = 0.779

## Discussion

This study shows for the first time that there is a strong interaction of predatory nematodes with nematodes as prey, both with and without substrate. Investigations into the type and strength of the interactions between predatory nematodes and their potential prey are important for a better understanding of the structure and function of the food webs in which these organisms participate. Our study of *P. muscorum* showed that it consumed large amounts of small and large *C. elegans*, with the daily *per capita* ingestion of prey reaching a maximum of 19.8 µg fresh weight, which corresponds to 4.8 times the predator’s biomass. In general, the number of ingested nematodes increased with an increasing number of prey offered, until a plateau was reached, regardless of prey body mass or habitat complexity, as shown in similar studies using other predators e.g.^16,22,34,35^. The proportion of biomass ingested vs. the predator’s own biomass was comparable to that reported for copepods^34^ and twice as high as that determined for tardigrades^22^. In the study of Nelmes^58^, adult individuals of the nematode *Prionchulus punctatus* were able to consume up to six times their own volume in nematodes per day. Previous studies have largely overlooked the potential impact of predatory nematodes on nematode communities, although early observations as well as our own suggest that the predation pressure on nematode prey provoked by predatory nematodes is an important factor in the modeling of food webs and calculations of carbon flow.

All four functional response curves of *P. muscorum* were type III and thus sigmoidal in shape, with a scaling exponent significantly greater than zero, as recently shown for several other predator- prey systems [e.g.^10–13,15,16^], and implying severe consequences for population dynamics and stability^11,59,60^. These results demonstrate that predator- prey body mass ratios together with environmental factors such as habitat complexity may significantly impact estimations of functional response parameters under laboratory conditions as well as food- web stability in natural ecosystems.

### Influence of prey size

Recent studies have shown a strong allometric effect on functional responses^13,14,21,61^. In the present work, the results of both functional response trials, i.e., with and without substrate, confirmed our initial hypothesis, that an increase in prey mass may cause a significant increase in handling time. Larger *C. elegans* are presumably harder to capture and process for *P. muscorum*, although observations of the predator’s feeding behavior in this study showed that prey of both size classes were swallowed completely. However, in some cases, *P. muscorum* individuals regurgitated large *C. elegans* several times before finally ingesting them. This behavior could explain the shorter handling times for small prey.

The values of the search coefficients were expected to follow a similar pattern, increasing with increasing prey body mass, because within a given area it is easier for a predator to encounter larger prey items^24,62^. For *P. muscorum*, however, this was only the case in the absence of substrate, as in the more complex habitat the search coefficients did not significantly differ between the two prey sizes, indicating that the predator’s advantage in capturing larger prey vanished in the presence of substrate. A sigmoid type III functional response was obtained for *P. muscorum* regardless of prey size and habitat complexity, but the scaling exponent was significantly higher for small prey without substrate. This result corroborated our hypothesis that increasing predator-prey body mass ratios are aligned with both higher capture rates and higher scaling exponents. As for the search coefficient, this effect could not be verified under conditions that included the presence of substrate, as the scaling exponents for the two prey sizes were essentially the same.

### Influence of habitat structure

Adding habitat structure to our experimental setup did not affect the functional response parameters for either prey size class as severely as reported by other authors^23,63^ and *per capita* consumption remained at comparable levels in the presence and absence of substrate. This may have been due to the nearly identical body shape and mobility of predator and prey, which allowed *P. muscorum* to follow *C. elegans* into almost all interstices, resulting in a low level of hideouts for the prey. A similar effect was described for the centipede *Lithobius mutabilis* feeding on springtails in leaf litter^23^. Nonetheless, contrary to our expectations, for large prey the search coefficient was not influenced by the presence of substrate and for small prey it was even positively affected. The addition of substrate to the experimental set-up may have created a three-dimensional environment that led to an improvement of the predator’s agility and enhanced its foraging behavior, at least for more easily handled small prey. Accordingly, the increase of the functional response curve for small prey with substrate was not as decelerated as without substrate with a significant lower scaling exponent. However, this scenario was ruled out by the results of the experimental trial with large prey, in which both the search coefficient and the scaling exponent remained unaffected.

Direct observations revealed the ability of *P. muscorum* to feed on nematodes much larger than those used in this study. These larger prey were discarded after being punctured or their contents were partially sucked out. Accordingly, subtle changes in prey size may dramatically alter both the feeding behavior and therefore the functional response of the predator. Other factors, such as habitat complexity, temperature and prey morphology, may exacerbate the impact of these changes^24^.

In conclusion, in our study of the relationship between a nematode predator and its nematode prey, the allometric effect of prey size had a greater effect on the predator’s functional response than did the addition of substrate, presumably due to the similar body shape and mobility of the two nematode species even though the difference between the two prey size classes used in this experiment was rather small. Our results demonstrate that individual factors, which may differ for every predator-prey relationship, are important determinants of functional responses and therefore potentially the ecosystem stability.

## Supplementary information

S 1

## Data Availability

The datasets generated and analysed during the current study are available from the corresponding author on reasonable request.
