# Supplementary material for: Effect of prey size and structural complexity on the functional response in a nematode- nematode system
Source: Sci Rep. 2019 Apr 5;9:5696. doi: 10.1038/s41598-019-42213-x (PMC6451004; doi:10.1038/s41598-019-42213-x)
Supplement: Supplementary file 1 — S 1 [file 41598_2019_42213_MOESM1_ESM.pdf]

Effect of prey size and structural complexity on the functional response in a nematode-nematode system

Bianca KREUZINGER-JANIK[1]*, Henrike BRÜCHNER- HÜTTEMANN[1] and Walter TRAUNSPURGER[1]

[1]*Bielefeld university, Animal Ecology, Konsequenz 45, 33615 Bielefeld, Germany*

*Corresponding author, e-mail: bianca.kruckemeyer@uni-bielefeld.de

S1 Overview of the experimental set-up of the functional response experiments without and with substrate and using small vs. large *C. elegans*, including the total tested prey densities and number of replicates (per treatment and in total).

substrate	without substrate				with substrate				
prey size	small prey		large prey		small prey		large prey		
treatment	control	predator	control	predator	control	predator	control	predator	
prey density	11	11	11	11	11	11	11	11	
replicates (n)	6	6	6	6	6	6	6	6	
total	66	66	66	66	66	66	66	66	**528**
